# Complex systems approach to scientific publication and peer-review system: development of an agent-based model calibrated with empirical journal data

**DOI:** 10.1007/s11192-015-1800-6

**Published:** 2015-12-10

**Authors:** Michail Kovanis, Raphaël Porcher, Philippe Ravaud, Ludovic Trinquart

**Affiliations:** INSERM U1153, 1 Place du Parvis Notre Dame, 75004 Paris, France; Université Paris Descartes – Sorbonne Paris Cité, Paris, France; Centre d’Epidémiologie Clinique, Hôpital Hôtel-Dieu, Assistance Publique-Hôpitaux de Paris, Paris, France; Cochrane France, Paris, France; Department of Epidemiology, Columbia University Mailman School of Public Health, New York, NY USA

**Keywords:** Peer review, Publishing, Computer simulation, Complex systems, Agent-based model

## Abstract

Scientific peer-review and publication systems incur a huge burden in terms of costs and time. Innovative alternatives have been proposed to improve the systems, but assessing their impact in experimental studies is not feasible at a systemic level. We developed an agent-based model by adopting a unified view of peer review and publication systems and calibrating it with empirical journal data in the biomedical and life sciences. We modeled researchers, research manuscripts and scientific journals as agents. Researchers were characterized by their scientific level and resources, manuscripts by their scientific value, and journals by their reputation and acceptance or rejection thresholds. These state variables were used in submodels for various processes such as production of articles, submissions to target journals, in-house and external peer review, and resubmissions. We collected data for a sample of biomedical and life sciences journals regarding acceptance rates, resubmission patterns and total number of published articles. We adjusted submodel parameters so that the agent-based model outputs fit these empirical data. We simulated 105 journals, 25,000 researchers and 410,000 manuscripts over 10 years. A mean of 33,600 articles were published per year; 19 % of submitted manuscripts remained unpublished. The mean acceptance rate was 21 % after external peer review and rejection rate 32 % after in-house review; 15 % publications resulted from the first submission, 47 % the second submission and 20 % the third submission. All decisions in the model were mainly driven by the scientific value, whereas journal targeting and persistence in resubmission defined whether a manuscript would be published or abandoned after one or many rejections. This agent-based model may help in better understanding the determinants of the scientific publication and peer-review systems. It may also help in assessing and identifying the most promising alternative systems of peer review.

## Background and significance

The burden associated with the worldwide scientific production has recently generated much debate and criticism about the sustainability of the established system of scientific publication. The exponential increase in number of manuscripts submitted for publication is much higher than the increase in number of researchers and overburdens the ability of available qualified referees (Ware and Mabe 2015; Gannon 2001; Laakso et al. 2011; Bohannon 2014; Arns 2014; Alberts et al. 2008). Peer-review resources are so scarce that recently the Nature Publishing Group experimented with outsourcing fast-tracked, paid peer review. Moreover, the associated costs are daunting. For example, for the UK higher education institutions alone, peer review would cost more than £110 million annually (Look and Sparks 2010). At the same time, a concern is that the peer-review system may be inefficient at detecting errors and even fraud (Hopewell et al. 2014; Bohannon 2013; Schroter et al. 2008; Stahel and Moore 2014). Most researchers believe that peer review is vital to scientific publication, but it needs to be improved to address all the challenges that arise (Mulligan et al. 2013; Nicholas et al. 2015).

Interventions to improve the system could be targeted to reviewers or the system itself. At the individual level, reviewers could receive special training or authors could be made aware of their identities. Rewarding peer reviewers could provide scientists with incentives to be more involved in peer review activities (Review rewards 2014). Interventions such as cascade peer review (passing reviews of rejected manuscripts to the next editor) or “crowd sourcing” of online reviews (the editor consults online comments along with the reviewers’ evaluations) could be implemented at the systemic level (Houry et al. 2012; van Rooyen et al. 1999; Patel 2014; M Ware 2013; Gura 2002; Stahel and Moore 2014; Cals et al. 2013). Assessing the impact of interventions to improve the system would require large-scale experiments, which are complex, costly and sometimes even impossible to perform. In fact, the available randomized controlled trials in the field are few (Rennie and Flanagin 2014).

Scientific publication and peer review need to be studied as a unified system, specifically as a complex system. Computer simulations can reproduce the complete behavior or even uncover data about some elements that are very difficult or impossible to be studied in real life. Agent-based models (ABMs) may be especially useful in this regard.

An ABM aims to simulate and reproduce the behavior and interactions of autonomous real-life agents. The agents interact with each other and their environment, for a complex behavior in the system that differs from the sum of the individual agent behaviors. The characteristics that drive agents’ behavior are stored in internal variables and are updated each time some specific conditions are fulfilled or at each time step (Auchincloss and Diez Roux 2008; Galea et al. 2010; Bonabeau 2002; Maglio and Mabry 2011; Epstein 2006). Agent-based modeling is an efficient way to study complex systems (Chhatwal and He 2015; Vespignani 2012; Farmer and Foley 2009; Marshall and Galea 2015; Alberts et al. 2008). It has been successfully used to reproduce and deal with real life problems, especially in public health (Rigotti and Wallace 2015). Previous pioneering studies have used ABM to simulate the peer-review system (F Squazzoni and Gandelli 2013; Paolucci and Grimaldo 2014; Nandi et al. 2013; Lee et al. 2010; Herron 2012; Allesina 2012; Day 2015; Park et al. 2014; Thurner and Hanel 2011).

We aimed to develop an ABM, by adopting a unified view of peer review and publication systems. We attempted to embrace the full complexity of the scientific publication system and use empirical data for calibration. We simulated all the interactions between authors, reviewers and editors and took into account the complete path of scientific manuscripts from submission to the final decision, including resubmissions, rejections after in-house review (without external peer review) and multiple rounds of peer review. We implemented the model in the biomedical and life sciences domain and used empirical data from medical journals for calibration. Our results closely match the real life situation.

Section “Collection of empirical data” of this article describes the sources for our data and section “Modeling the scientific publication and peer-review system”, how scientific publication works in real life and the development of our ABM. Section “Calibration procedures and main outputs of the model implementation” describes how we parameterized submodels so that the ABM outputs reproduced the real-life data, and section “Sensitivity analyses” provides the results of our model and sensitivity analyses.

## Collection of empirical data

To guide the development and parameterization of the ABM and to perform calibration procedures, we collected empirical data pertaining to the medical domain. We contacted a sample of medical journals to obtain their acceptance rates, with a 40 % positive response rate. We consulted journal websites to obtain the remaining acceptance rates (when available). Finally, we collected the journal impact factors from Journal Citation Reports 2013 and the remaining data from a previously published international survey (Mulligan et al. 2013).

### Characterization of journals: survey of editors

Among 119 journals indexed in the MEDLINE Core Clinical Journals subset, we surveyed 105. We excluded journals that invited only submissions (*n* = 11) and those no longer active (*n* = 2); a journal’s special edition was considered among the regular issues.

We searched the website for each journal for the contact details of the editor-in-chief or editorial office. On December 5, 2014 we sent an email asking for the number of manuscripts submitted to the journal in 2013, number of manuscripts rejected after in-house review (without external peer review) and number of articles published in 2014. We sent 2 reminders on December 12 and January 22, 2015 and closed our survey on February 1, 2015. We masked the data so that journals could not be matched to their acceptance rates.

We had a response rate of 68 and 40 % for the 105 journals finally provided us with data. For journals that did not provide data, we searched their websites for reported acceptance rates and estimated the number of published articles for 2014 from the Journal Citation Reports 2013. Finally, we collected the acceptance rates for 62 journals and rejection rates after in-house review of 45 journals. We obtained the impact factors for each journal from the Journal Citation Reports 2013 and rescaled them for standardization (Table 1).

**Table 1:** Data from MEDLINE Core Clinical Journals

	Data for 2013
Rescaled impact factor(*n* = 105)	0.11 ± 0.14 [0.0–1.0]
Acceptance rate (*n* = 62)	0.22 ± 0.11 [0.43–0.59]
Rejection rate after in-house review (*n* = 45)	0.37 ± 0.22 [0.00–0.81]
No. of submissions (*n* = 105)	173,436
No. of rejections after in-house review (*n* = 105)	52,373
No. of published papers (*n* = 105)	32,729

Data are mean ± SD [min–max] from a survey of journal websites and the Journal Citation Reports 2013

### Characterization of system processes

We used data from the international survey conducted by Mulligan et al. (2013). The authors contacted 40,000 researchers and obtained 4037 responses. We obtained data for the “medicine and allied health and nursing” domain (565 researchers) for time to final decision for a manuscript (Table 2B) and number of articles that researchers had published (Table 2C). Finally, by directly contacting authors we obtained also the data for the number of submissions up to publication (Table 2A).

**Table 2:** Empirical data characterizing the system processes

Process	
A. No. of submissions until publication	No. of articles (*n* = 565) (%)
1	15
2	47
3	23
4	12
More than 5	4

B. Time to final decision	No. of articles (*n* = 504) (%)
≤1 week	1
2–3 weeks	5
1–2 months	19
3–6 months	49
>6 months	25

C. Articles	Researchers (*n* = 4037) (%)
1–5	14
6–10	13
11–20	18
21–50	26
51–100	18
>100	11

Data from Mulligan et al. (2013) international survey and from personal contact with authors

## Modeling the scientific publication and peer-review system

### Description of the system

**Fig. 1:**
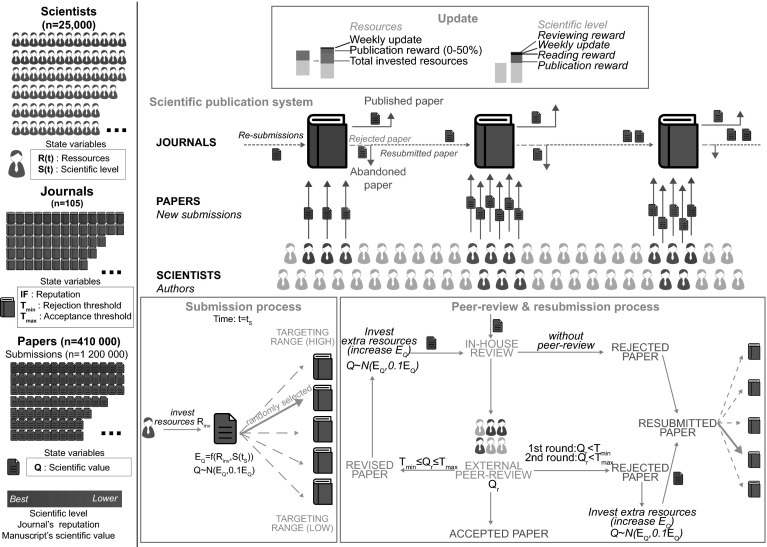
Description of the agent-based model. The agents and the processes by which our agent-based model operates. Key features are the submodels of the submission and the peer-review process

Scientific publication in its most typical form can be described as a complex system in which researchers interact with each other taking the roles of authors, journal editors and reviewers (Fig. 1) (Brown 2004). Researchers conduct research by using many resources (e.g., grants, research facilities or collaborations). They promote their findings and make them available to the scientific community by reporting them in scholarly manuscripts, which they submit to scientific journals for publication. Decisions on publication are based on multiple factors including the paper’s quality, novelty, importance or controversy.

Journals first perform an in-house review to determine whether they will reject a manuscript immediately (e.g., irrelevant to a journal’s scope or below quality standards) or send the manuscript for external peer review. In-house review commonly involves the editor-in-chief and members of the editorial board. For the external peer review, the editor solicits external researchers to review articles. On the basis of the editor’s and external peer-reviewers’ assessments, the editor decides to accept the paper, ask for revision (acceptance is not guaranteed) or reject the manuscript. Revisions require a second or further round of peer review (Wilson 2012). Rejected manuscripts may be resubmitted to other journals or ultimately be abandoned and remain unpublished. Published articles, depending on their impact on the scientific community, help researchers obtain additional resources. Moreover, researchers benefit from reviewing scientific manuscripts in terms of knowledge.

### Agent-based model

We modeled researchers, manuscripts and journals as agents of the scientific publication system from the interactions of their respective state variables (Fig. 1). The researchers could be both authors and reviewers, but editors and journals were modeled as the same agent. The ABM is organized in submodels. The ABM is organized in submodels. Each of the submodels can be parameterized independently. Some submodels pertain to the submission process, including the creation of manuscripts and the targeting of journals for the first submission. Others pertain to the peer review process, including peer review rounds and resubmissions.

### Researchers

We characterized *N* researchers by two state variables: resources *R*(*t*) and scientific level *S*(*t*) (Squazzoni and Gandelli 2013). The scientific level was defined as *S*(*t*) = *R*(*t*) + *S*_*b*_(*t*), where *t* the time step and *S*_*b*_(*t*) the sum of all the rewards that a researcher can receive to determine scientific level, as explained at the end of this section. The resources represent all the means that researchers have at their disposal for conducting research. The scientific level expresses a researcher’s experience and capacity to conduct better research. In our model, scientific knowledge evolves by a researcher’s own research (published articles), the evolution of resources, and from reading and reviewing other manuscripts.

**Fig. 2:**
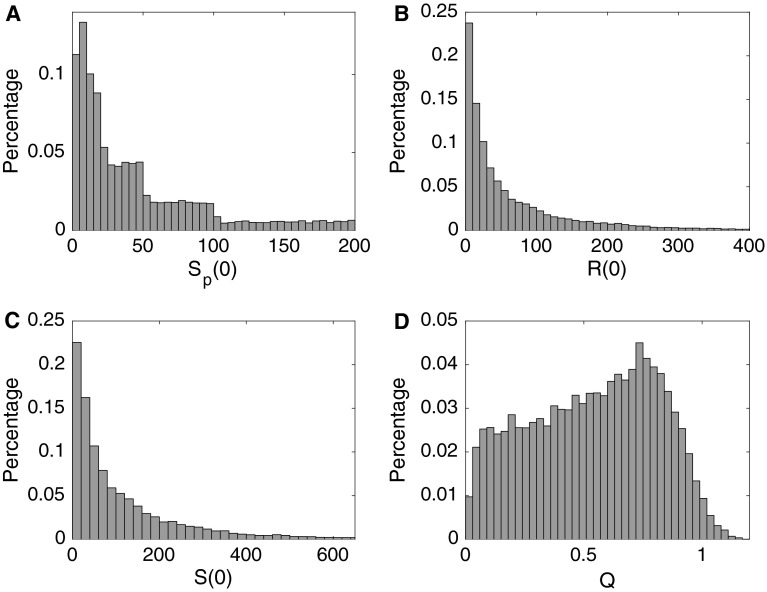
Distribution of the initial state variables of researchers and articles. Distribution of **a** initial number of published articles per researcher *S*_*p*_(*t* = 0), **b** initial amount of resources per researcher *R*(*t* = 0), **c** initial scientific level per researcher *S*(*t* = 0), and **d** manuscript scientific values (*Q* scores) when all researchers (*N*) hypothetically invest half of their available resources at time *t* = 0

For *t* = 0 we set *S*_*b*_(0) = *S*_*p*_(*t*), where *S*_*p*_(*t*) is the cumulative amount of publications per researcher at time *t*. We initialized *S*_*p*_(0) (Fig. 2a) using the empirical distribution in Table 2C and set *R*(0) = *γS*_*p*_(0) (Fig. 2b) where *γ* was uniformly distributed over 0.1 and 3. The initial distribution of *S*(0) can be seen in Fig. 2c.

### Manuscripts

Manuscripts were characterized by the state variable *Q*, which serves as a proxy for their intrinsic scientific value but also their disruptive, innovative, or controversial nature as well as quality of reporting. At each time step, *N*_*s*_ randomly selected researchers submitted their paper (as detailed in the Calibration section). At the time of submission *t*_*s*_ of their paper, authors would lose an amount of resources *R*_inv_ associated with the conduct of the research reported in that paper—$0.2R(t_{s} ) \le R_{\text{inv}} \le 0.7R(t_{s} )$. However, for researchers with resources, we set *R*(*t*_*s*_) < *R*_min_ = 1 so that they could not submit any work for publication and had to wait until they obtained more resources.

Each paper had an initial expected quality *E*_*Q*_ defined by both the amount of resources the author invested and the author’s scientific level at *t*_*s*_ (F Squazzoni and Gandelli 2013)

$$E_{Q} = 0.8\frac{{0.1R_{\text{inv}} }}{{0.1R_{\text{inv}} + 1}} + 0.2\frac{{0.01S\left( {t_{s} } \right)}}{{0.01S\left( {t_{s} } \right) + 1}}$$

The *Q* score was drawn from a normal distribution $Q - N \left( {E_{Q} , 0.1E_{Q} } \right)$. This score determines how a researcher chooses a target journal and drives in-house and external peer-review assessments. If all researchers invested half of their initial resources at *t*_*s*_ = 0 to create manuscripts, then the distribution of *Q* scores would be as seen in Fig. 2d.

### Journals

**Fig. 3:**
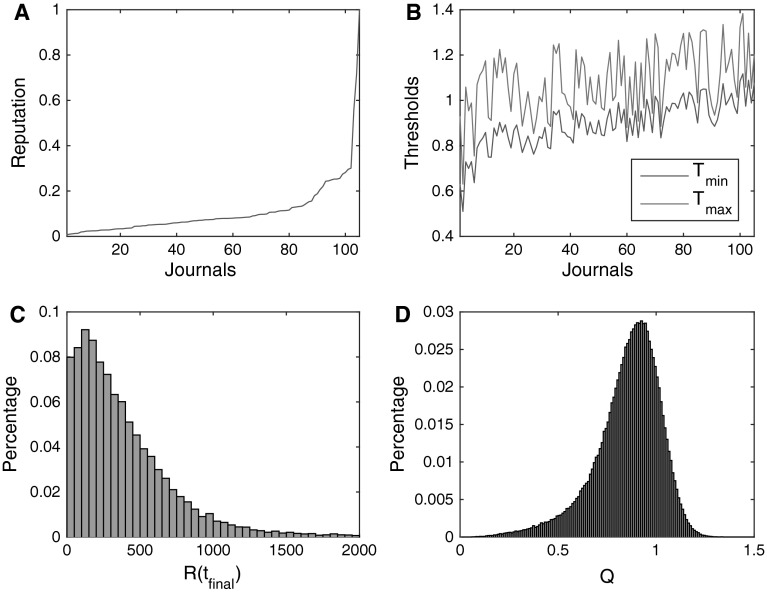
Distribution of the final state variables of researchers, journals, and manuscripts. Distribution of the **a** journal reputation derived from the rescaled impact factors for 2013, **b** journal rejection and acceptance thresholds $\left( {T_{\hbox{min} }^{j} ,T_{\hbox{max} }^{j} , j = 1, \ldots , J} \right)$, **c** resources per researcher at the end of the simulations [*R*(*t* = 520)], and **d** manuscript final scientific values (*Q* scores)at the end of simulations

We characterized *J* journals by three state variables: a reputation value [we used rescaled impact factors (Fig. 3a)] and by related rejection or acceptance thresholds, $T_{\hbox{min} }^{j} < T_{\hbox{max} }^{j} , \;j = 1, \ldots , J$ (Fig. 3b). The reputation and thresholds were used to define how a researcher chose a target journal and if a manuscript was rejected or accepted after in-house or external peer review.

The rejection or acceptance thresholds reflected the ranking of journals by their reputation and were defined by the expected scores of submissions journals receive. For each year, we drew *N* score values for a fictitious sample of upcoming submissions; we estimated the *J*-quantiles *q*^*j*^ of this distribution, including the minimum value, and defined $T_{\hbox{min} }^{j} = \delta_{\hbox{min} } q^{j} + n^{j}$ and $T_{\hbox{max} }^{j} = \delta_{\hbox{max} } {\rm T}_{\hbox{min} }^{j} + n^{j} - C$, where $\delta_{\hbox{min} } ,\delta_{\hbox{max} }$, and *C* were constants and *n*^*j*^ was random (as detailed in the Calibration section). This definition kept the distribution of acceptance rates insensitive to changes in the distribution of resources.

### Journal targeting process

To define how a researcher chose a target journal, we assumed that authors had a general knowledge of journal standards and, given the score, would try to obtain the most recognition from their work. Hence, the journal for the first submission was chosen at random among those with $T_{\hbox{min} }^{j}$ within the asymmetrical range $Q - 0.45 \varepsilon \le T_{\hbox{min} }^{j} \le Q + 0.55\varepsilon$, where $\varepsilon - 2 \times N \left( {\frac{Q}{5},\frac{Q}{20}} \right)$. This process resulted in a slight trend of high targeting in every first submission.

### In-house and external peer-review process

We drew the editor’s assessment of the manuscript *Q*_*e*_ from a uniform distribution over $\left[ {0.9Q;1.1Q} \right]$. If $Q_{e} < T_{\hbox{min} }^{j}$, the manuscript could be rejected without external peer review, depending on the journal’s reputation; the likelihood of editorial rejection was larger for journals with larger reputation (as detailed in the Calibration section). If $Q_{e} \ge T_{\hbox{min} }^{j}$, the manuscript was sent for external peer review; two or three reviewers (with 20 % probability) were randomly selected to their scientific level and the journal’s reputation; the top 10 % journals randomly select reviewers among the top 10 % researchers and so on. The reviewers’ assessments were defined as $Q_{r} - N \left( {Q - c,r \times Q} \right)$, where *r* was a random error and *c* measured the competitiveness of the reviewer.

The error factor *r* represents the reliability of the reviewer’s assessment. It depended on the amount of time the reviewer spent evaluating the manuscript, the reputation of the journal and the score of the manuscript itself. We assumed that the more time spent on the assessment, the greater the reputation of the journal, and the greater the score, the greater the chance of an accurate assessment. Formally, we defined *r* = *r*_*r*_ + *r*_*j*_ − *r*_*Q*_, where *r*_*r*_ is the reviewing error, *r*_*j*_ the journal error and *r*_*Q*_ the score error. With 65 % probability, we set *r*_*t*_ = 0.1; with 12 %, *r*_*t*_ = 0.05; and with 13 %, *r*_*t*_ = 0.01 We drew *r*_*j*_ randomly from a uniform distribution over [0; 0.15], where *r*_*j*_ = 0 corresponded to the highest reputation journal and *r*_*j*_ = 0.15 to the lowest. Finally, *r*_*Q*_ = 0.05 × *Q*.

The competitiveness factor *c* depended solely on the reputation of the journal and represents potential reviewer conflict of interest affecting the assessment of the manuscript. We assumed that a competitive behavior would occur more often for journals with higher reputation. The probability of appearance ranged uniformly from 10 to 66 %, where *c* was drawn randomly from a uniform distribution over [0.01; 0.05].

We randomly selected one of the reviewers’ evaluations as a proxy of the editor’s opinion. We simulated more than one reviewer to be able to update their scientific levels appropriately. If *Q*_*r*_ ≥ *T*_max_, the manuscript was accepted and if *Q*_*r*_ ≤ *T*_min_, it was rejected. When *T*_min_ ≤ *Q*_*r*_ < *T*_max_, the author was asked to revise the manuscript before a second round of peer review.

In the later case, the author invested an extra amount of resources $R_{\text{imp}} - N\left( {\frac{8}{60},\frac{1}{60}} \right) \times \left( {R - R_{\text{inv}} } \right)$. The cumulative amount of invested resources was used to derive a new *Q* score as before. The manuscript was re-evaluated by two or three reviewers, randomly selected again, and accepted only if *Q*_*r*_ ≥ *T*_max_. The *Q*_*r*_ from the second round of peer review was calculated only from the randomly chosen evaluation from the two or three new reviewers.

Following a rejection after in-house review or external peer review, an author could resubmit the manuscript.

### Resubmission process

The probability of resubmission *P*_res_ after a rejection decreased with increasing number of resubmissions *r* increases, $P_{\text{res}} = P_{0}^{r - 1}$. The *P*_0_ value was defined with the calibration procedure.

If a manuscript was rejected after external peer review, we assumed that the authors could substantially revise it by investing extra resources $R_{\text{imp}} - N\left( {\frac{20}{60},\frac{2}{60}} \right) \times \left( {R \left( {t_{s} } \right) - \left( {R_{\text{inv}} + \sum\nolimits_{i} {R_{\text{imp}}^{i} } } \right)} \right)$, where *R*(*t*_*s*_) are the resources before at the time of submission and *i* the times the author invested extra resources to improve it. If a manuscript was rejected after in-house review, we assumed that authors invested a smaller amount of extra resources $R_{\text{imp}} - N\left( {\frac{1}{60},\frac{0.1}{60}} \right) \times \left( {R (t_{s} ) - \left( {R_{\text{inv}} + \sum\nolimits_{i} {R_{\text{imp}}^{i} } } \right)} \right).$

We assumed that after a first rejection, the authors would target journals of lower reputation than for the first submission. Thus, they randomly selected journals in the (symmetrical this time) range $pQ - 0.5\varepsilon \le T_{\hbox{min} }^{j} \le pQ + 0.5\varepsilon$, where *Q* is the initial score of the manuscript and 0 < *p* < 1 the targeting of lower reputation journals. This rule allowed for easier acceptance after the second submission, because the score of the manuscript was >*pQ* after resubmission.

### Duration of the peer-review process

For estimating the duration of the peer-review process from submission to final decision, we used the distribution from Table 2B. We assumed that rejection after in-house review occurred within 3 weeks, whereas decisions after one or more rounds of external peer review occurred after ≥1 month. When a manuscript is accepted, it takes an extra 1–2 months for publication. Resubmissions occur instantly as the final decision is announced.

### Updating of variables

Resources and scientific level were updated at each time step. Resources invested for conducting and reporting research *R*_inv_ were subtracted at the time of initial submission, whereas the extra resources *R*_imp_ were subtracted uniformly until the time of a journal’s final decision. Thus, a researcher allocated resources to both new research manuscripts and already (re)submitted manuscripts. If the article is published, the author received a reward between 0 and 50 % of the total amount of invested resources, $p \times \left( {R_{\text{inv}} + \sum\nolimits_{i} {R_{\text{imp}}^{i} } } \right),0 \le p \le 0.5$. If a manuscript remained unpublished, the author would permanently lose all the resources invested.

The scientific level *S*(*t*) = *R*(*t*) + *S*_*b*_(*t*) evolved according to resources and number of published or reviewed manuscripts. In case of publication, the author received a reward for resources in scientific level together with an increase in the number of publications *S*_*P*_(*t*). The extra resources invested for revisions were subtracted uniformly from the scientific level until the time of the final decision. The scientific level of a reviewer was credited with a random reward between 0 and 0.001 every time the reviewer completed a review because of knowledge acquired from the paper. Moreover, the scientific level of all researchers was credited with a reward at each time step to reflect the impact of newly published articles, drawn from a normal distribution *N*(*I*, 0.1*I*), where *I* is the average across all articles published the previous week of $0.1Q_{\text{final}} \times IF_{\text{final}}^{j}$ (i.e., the quality score of a published article × the impact factor of the journal that published it). The greater the article quality score and journal impact factor, the higher the chance a researcher would read the article and gain knowledge from it and the larger the reward. Finally, at the end of each week, the researchers received an update to their resources and scientific levels randomly drawn between 0.1 and 1, which reflected an increase of the means to conduct research with time.

## Calibration procedures and main outputs of the model implementation

We programmed the model using MATLAB (MATLAB and Statistics Toolbox Release 2014b, The MathWorks, Inc., Natick, Massachusetts, United States). The code is available at http://www.clinicalepidemio.fr/peerreview_abm/. We programmed the model with a total population of researchers *N* = 25,000 and total population of journals *J* = 105. We ran the simulations for 10 years, with a burn-in period of 1 year for the initialization of the model. Results were averaged over 20 simulations. The main outputs measured were total number of publications per year, proportion of successfully published articles compared to all submissions, proportion of manuscripts revised before being published and proportion of manuscripts for which the peer review process improved their *Q* score after revision.

We developed our ABM so that its mechanisms resembled those operating in the real-life scientific publication system. We parameterized the model by calibration procedures so that it fit empirically observed data. We considered that this assumption was verified if the model achieved good fit for the distribution of acceptance rates, rejection rates after in-house review, number of submissions until publication, and yearly number of published articles. Goodness-of-fit was assessed by the Anderson–Darling test *p* values across all runs; we report the minimum and maximum *p* values.

### Distribution of acceptance rates after external peer review

**Fig. 4:**
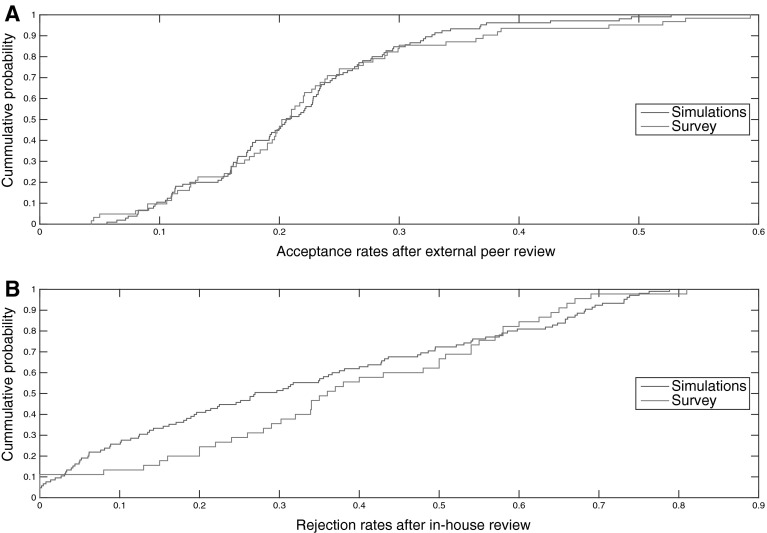
Calibration of acceptance rates after external peer review and rejection rates after in-house review. Empirical cumulative distribution functions of **a** acceptance rates after external peer review for empirical and simulated data (single run). The model output fits the empirical distribution [Anderson–Darling *p* values (0.63–0.73) in all runs] and **b** rejection rates after in-house review for empirical and simulated data (single run). The model output fits the empirical distribution [Anderson–Darling *p* values (0.068, 0.152) in all runs]

We sorted journals in ascending order by reputation. We generated $n^{(j)} = u^{j} - z_{1}^{(j)} + F \times z_{2}^{(j)}$, with $u^{j} - U\left( {0.01,0.20} \right)$, $z_{1}^{j} - N\left( {0,0.45} \right)$ and $z_{2}^{j} - N\left( {0,0.015} \right)$ and $n^{(j)} ,z_{1}^{(j)}$ and $z_{2}^{(j)}$ order statistics; *F* = 1 for the 20 % highest reputed journals and *F* = 0 for the others. We set $T_{\hbox{min} }^{j} = 0.9q^{j} + n^{j}$ and $T_{\hbox{max} }^{j} = 1.2{\rm T}_{\hbox{min} }^{j} + \left( {n^{j} - 0.095} \right)$. We obtained an acceptance rate of 0.21 ± 0.09, which is almost identical to the one obtained from the survey. Figure 4a shows that the model output fits the empirical distribution of acceptance rates (Anderson–Darling *p* values [0.63–0.73]).

### Distribution of rejection rates after in-house review

The empirical distribution of rejection rates after in-house review was uniformly spread across impact factors, except for a peak at zero, corresponding to journals that send all submissions for external peer review. To calibrate the distribution, we defined the strictness of the journals as a probability, a linear function of their reputation $\Pr_{e}^{j} = \frac{j}{105}$. We also assumed that 20 % of the highest ranked journals would be strict with their editorial policies and would reject everything <$T_{\hbox{min} }^{j}$, whereas five of them—excluding the 10 % with the highest reputation—would send everything for external peer review (according to survey data).

Therefore, rejections after in-house review would occur only if $Q_{e} < T_{\hbox{min} }^{j} \;{\text{and}}\;\Pr < \Pr_{e}^{j}$, where 0 ≤ *Pr* ≤ 0.8 is a random number drawn from a uniform probability distribution. We randomly selected five journals that sent everything for peer review (excluding the top 10 % journals with the highest reputation). This process resulted in uniformly distributed rejection rates after in-house review that match the empirical data as seen in Fig. 4b (mean value [0.32 ± 0.25] and Anderson–Darling *p* values [0.068, 0.152]).

### Number of submissions until publication

To calibrate the distribution of submissions until publication, we set *p* = 0.68, so that authors target journals in the range $0.68Q - 0.5\varepsilon \le T_{\hbox{min} }^{j} \le 0.68Q + 0.5\varepsilon$ when resubmitting and *P*_0_ = 0.88. The results in Table 3 show that the ABM outputs fit the empirical data well.

**Table 3:** Comparison of distribution of resubmissions (survey vs agent-base model)

Resubmissions	International survey (%)	Agent-based model (%)
0	14.6	14.89 ± 0.09
1	46.9	47.21 ± 0.22
2	22.6	20.35 ± 0.11
3	11.7	9.41 ± 0.09
4	2.4	4.46 ± 0.05
5	0.5	2.095 ± 0.022
6	0.6	0.94 ± 0.04

Data from Mulligan et al. (2013) international survey and from personal contact with authors

### Total publications per year

**Fig. 5:**
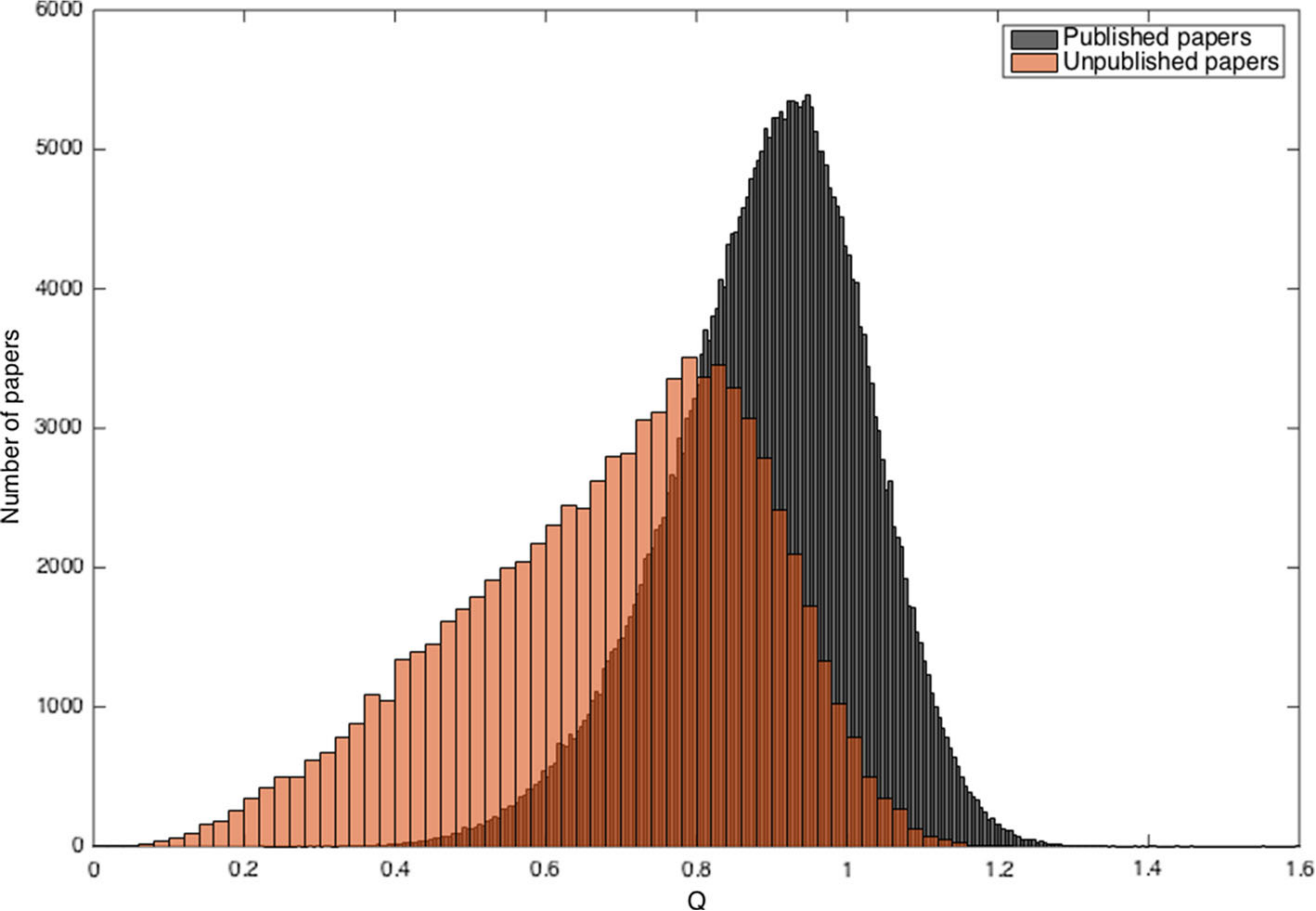
Distribution of scientific values (*Q* scores) of published and unpublished articles. 81 % of the total submissions were finally published, with their average ***Q*** score 29 % higher from the average ***Q*** score for unpublished manuscripts

For each week, we randomly selected $N_{s} - N\left( {800,80} \right)$ authors to invest resources and create manuscripts. The authors produced 33,598 ± 203 manuscripts per year as compared with the 32,729 manuscripts estimated from the empirical data for 2013. From these, 87 % were revised before publication and for 75 % of these, the quality was improved as compared with the empirical values of 92 and 88 %, respectively (Mulligan et al. 2013). Overall, 81 % of the total submissions were finally published, with their mean *Q* score 0.89 ± 0.13, whereas those unpublished had a mean *Q* score 0.69 ± 0.20; a relative difference of 29 % (Fig. 5).

### Main outputs

Our model fulfills stationarity and ergodicity, and thus, results from a single run do not differ significantly from the average for several runs. We present the results for the main outputs of the model across 20 simulation runs in Table 4.

**Table 4:** Main outputs of the model and their deviations across 20 simulation runs

Yearly publications	Published manuscripts	Revised manuscripts	Improved manuscripts after revisions	Score of published articles	Score of unpublished articles
33,598 ± 203	81.00 ± 0.22	86.69 ± 0.09	75.13 ± 0.11	0.8950 ± 0.0007	0.6880 ± 0.0018
[33,244–34,021]	[80.00–81.00]	[86.57–86.88]	[74.91–75.31]	[0.8940–0.8968]	[0.6855–0.6919]

Data are mean ± SD, in the first row, and [min–max], in the second row, across all simulation runs. We ran the simulations for 10 years, with a burn-in period of 1 year for the initialization of the model, and averaged the results for 20 simulation runs. All runs were calibrated and the outputs varied slightly between the runs

## Sensitivity analyses

We performed two types of sensitivity analyses. First, we selected four variables central to the structure of the model parameters and explored a broad range of values for each so that we could evaluate how they affect the outputs of the ABM. We then performed an extra simulation whereby we evaluated the synergy of the parameter values that maximized difference in the average scores of the published and unpublished manuscripts. Second, we explored various scenarios that incorporate changes in the initial targeting strategy of the authors and in the reviewing behavior of the referees. We compared the results with the standard case for each, to better understand how initial targeting and reviewing strategies can affect the model outputs.

### Parameter variation

We performed a sensitivity analysis of the four variables central and varied the targeting when resubmitting (**p**), the volume of weekly submissions (**N**_**s**_), the probability of resubmission (**P**_0_), and the strictness of the in-house reviewing policy (**Pr**_**max**_). We measured the impact of the variables on the yearly amount of publications, the proportion of published articles and the difference in the mean scores of published and unpublished manuscripts. For simplicity, we refer to this difference as “score gap”.

The scaling of **N**_**s**_ linearly increased the value of all the three measured outputs. However, variations in the value of **Pr**_**max**_ did not have any notable impact on the outputs. The variation of **p** produced the highest difference in the score gap; 40 % relative increase compared to the correct calibration. No parameter variation decreased the difference <10 %. High values of **p** and low values of **P**_0_ decreased the amount of yearly publications and the percentage of published manuscripts, and vice versa. All results are shown in Table 5.

**Table 5:** Sensitivity analyses defined by varying four parameters central to structure of the model

Parameter descriptions	Parameter names	Range of variation	Step of variation	Yearly publications	Published manuscripts (%)	Score gap
Targeting when resubmitting	*p*	[0.9–0.1]	−0.1	[25,911–36,057]	[62–86]	[0.19–0.28]
Volume of weekly submissions	*N*_*s*_	[400–2000]	200	[16,086–86,131]	[78–83]	[0.18–0.27]
Probability of resubmission	*P*_0_	[0.55–0.95]	0.05	[27,459–35,926]	[66–87]	[0.19–0.23]
Strictness of the in-house reviewing policy	Pr_max_	[0.1–0.9]	0.1	[33,170–33,770]	[80–81]	[0.20–0.21]

Range of desired outputs [min–max] from sensitivity analysis. The variation of *p* is presented as a max to min value, because the highest value of *p* corresponds to the lowest output results and vice versa. Pr_max_ did not substantially affect the outputs, whereas *N*_*s*_ affected them linearly. The variation in *p* produced the highest score gap (+40 % compared to the correct calibration)

We performed an extra simulation round for evaluating the extreme scenario, whereby we parameterized the ABM with the values of *p* and *P*_0_ that produced the maximum score gap. We did not re-parameterize **Pr**_**max**_, because variations of its value did not substantially affect the outputs, and **N**_**s**_, because performing simulations for the same amount of scientists submitting per week more than 150 % manuscripts than in the calibrated case would be unrealistic.

The values that affected the score gap the most were *p* = 0.5 and *P*_0_ = 0.95 (+40 and +15 % compared to the correct calibration, respectively) and inputted in the model for performing the extra simulation run. The new, more persistent but less ambitious, behavior of the authors when resubmitting resulted in a 55 % increase in the score gap. This increase was produced mainly from the decrease in mean score of the unpublished manuscripts, so with this re-parameterization, the ABM was more capable of low *Q* score at the screening of papers.

### Simulation scenarios

We considered the standard and two additional targeting strategies. In the first strategy, scientists initially submit to journals of lower rejection threshold than they do in the standard case ($Q - 0.65\varepsilon \le T_{\hbox{min} }^{j} \le Q + 0.35\varepsilon$). In the second strategy, they target journals of higher rejection threshold ($Q - 0.35\varepsilon \le T_{\hbox{min} }^{j} \le Q + 0.65\varepsilon$). For each of the three strategies, we also considered two additional reviewing scenarios and the standard reviewing scenario. The first scenario assumed that the reviewers would be competitive only if the manuscript they currently review has the same or up to 5 % score as their last published paper. Then they would randomly evaluate its average score as being 5–10 % lower, with all other reviewing errors remaining the same. The second scenario assumed that all evaluations, both of reviewers and editors, are accurate, with no errors.

We compared eight scenarios with the standard model to evaluate how targeting and reviewing affects the system in terms of average number of resubmissions before publication, improvement in papers’ scores and increase in the gap in the average scores between published and unpublished papers. No scenario raised the percentage of improved papers after peer review more than 3 %. The fair reviewing strategy increased the score gap the most in all cases (15–16 %), and the rest produced changes varying from −1 to 2 %. Considering the amount of average resubmissions, the competitive case resulted in a decrease ranging from 8 to 15 %, whereas changes from the fair case were insignificant (<5 %). Results in Table 6.

**Table 6:** Sensitivity analyses defined by the simulation of certain additional scenarios

Initial targeting	Reviewing strategy	Average resubmissions	Improvement after peer review (%)	Average score gap	Relative score gap (%)
Low	Competitive	1.41	74	0.21 [0.69, 0.90]	2
Low	Fair	1.51	78	0.24 [0.67, 0.91]	16
Low	Standard	1.50	75	0.21 [0.69, 0.89]	0
Standard	Competitive	1.46	74	0.21 [0.69, 0.90]	**−**1
Standard	Fair	1.53	78	0.24 [0.67, 0.91]	15
Standard	Standard	1.56	75	0.21 [0.69, 0.89]	N/A
High	Competitive	1.48	74	0.20 [0.69, 0.90]	**−**1
High	Fair	1.57	74	0.24 [0.67, 0.91]	16
High	Standard	1.56	75	0.20 [0.69, 0.89]	**−**1

In this table we see the outputs of the eight scenarios and the relative score gap as compared to the calibrated model [standard–standard]

## Discussion

Our ABM mimics the properties and functions and addresses different scenarios of behavior and interactions in the scientific publication system. The main strengths of our model are the use of empirical data, which allowed us to produce realistic outputs, and the unified view of evaluation and publishing systems. The main difficulty in the calibration was that we had to reproduce the whole journey of a manuscript from its submission to publication or until the authors give up on submitting it. From empirical data from the biomedical and life sciences domain, we calibrated the model so that the journals do not accept or reject too many manuscripts and so that the manuscripts are not resubmitted more than is required, before being published.

We obtained an amount of publications very close to that estimated from our survey. This situation allowed us to examine characteristics of manuscripts that remained unpublished and were handled inside the ABM with realistic rules and calibration. In all, 19 % of submissions that received a final decision were never published, and their mean *Q* score significantly differed from that for published articles. A moderate proportion of unpublished manuscripts had *Q* scores close to the high scores of the published articles. This issue is a problem of the scientific publication system, in which editors may sometimes make questionable gatekeeping decisions (Siler et al. 2015). The reasons why an unpublished manuscript considered worthy of publication was not published include poor targeting, mistakes in the in-house or external peer review and lack of persistence in resubmitting the manuscript.

From our sensitivity analyses, variations in the strictness of journals’ editorial policies were not able to significantly affect the system. Changes in the amount of weekly submissions linearly affected the model outputs. Behavioral changes in the resubmission strategies of the authors could significantly affect the distribution of *Q* scores of the unpublished manuscripts. The synergetic effect of lower targeted and more persistent resubmissions increased the difference in average scores of published and unpublished papers by 55 %. This finding suggests that the system can publish more papers of higher *Q* score by changes in the resubmission attitudes of authors. However, for producing significant changes in other parts of the system, one needs to consider alternative interventions that will come from structural changes in how the journals and the whole system functions.

From the eight different scenarios, we found that the alternative competitive behavior we introduced reduced the average resubmissions until publication by 8–15 %, without affecting the amount of published articles. The fair scenario produced the highest relative difference in the score gap (15–16 %), which was independent of the initial targeting strategy. However, this difference is still lower than the score gap produced by modifying only the authors’ resubmitting behavior. Also, in all cases, the score gap increased or decreased because of the average *Q* score of the unpublished distribution. Finally, the percentage of papers that benefited from peer review did not deviate more than 3 % compared to the standard case for any of these scenarios.

Specific aspects of the peer-review system have previously been studied by pioneering works using ABM approaches (Squazzoni and Gandelli 2013; Park et al. 2014; Allesina 2012; Day 2015; Herron 2012; Paolucci and Grimaldo 2014; Thurner and Hanel 2011). Squazzoni and Gandelli (2013) modeled a system whereby authors and reviewers interact in the environment of a single journal. They simulated three different scenarios; in the first scenario, the reviewers reciprocated the behavior of previous reviewers towards them; in the second scenario, the reviewers’ behavior was not affected by past actions and in the final scenario, the reviewers were reciprocating fair evaluations of their papers. The authors’ results suggest that reciprocity can benefit peer review only when inspired by disinterested standards of fairness (Squazzoni and Gandelli 2013). Paolucci and Grimaldo (2014) replicated the results of Thurner and Hanel (2011) by using a “redesign” approach. In their approach Scientists, Conferences and Papers interact, whereas reviewers can follow different types of reviewing strategy (Correct or Rational Cheating). The authors show that the obtained results are fragile to small mechanism variations and suggest that exploration at the level of mechanisms is necessary for supporting theoretical statements with simulations (Paolucci and Grimaldo 2014). Allesina (2012) modeled a “classical” setting of the scientific publication system—using 50 journals and 500 researchers—and compared it in terms of efficiency to two alternative settings of the system: “editorial rejection”, in which editors could reject manuscripts after in-house review and “bidding”, in which authors submit their paper to a pool of manuscripts and journals bid for them. The “editorial rejection” setting raised the publication speed, decreased the burden to the reviewers and provided better control for quality but raised the rejection rates and the probability of Type I errors. The “bidding” setting provided faster publication, better distribution of peer review effort and more publications for authors in better journals although with higher probability of Type II errors and more burden to the editors (Allesina 2012).

However, a holistic approach to evaluate the entire scientific publication system, using empirical data, had not been attempted. Previous studies focused solely on peer review, only a part of our model, or they did not address the full complexity of the system (e.g., large scale of the system, multiple rounds of peer review or revisions of manuscripts after peer review). Despite the continual “risk of brutal oversimplification”, we attempted to address the full complexity of the system on a large scale and incorporate empirical data to calibrate its processes (Squazzoni 2010). A reliable base model that better characterizes the standard system must be built and then alternatives to this standard system constructed by comparison because the robustness of inference about the comparison will be influenced by how the standard system is adequately captured by the base model.

The calibration alone was complex, but it was important for describing accurately the base system. We achieved the calibration by “fine-tuning” some microscopic variables to fit empirical data for a limited number of strategically chosen parameters. Alternative systems can be incorporated in the model by making structural changes to some of its submodels. This inclusion will consequently affect the macroscopic outputs. An alternative system would be to crowdsource online reviews and use it along the standard peer review. For implementing this, we need to make additions and modifications to the structure of the submodels of the peer-review process, keeping every other relation and value the same. A structural change could be to allow randomly selected scientists to provide evaluations for a paper, as a form of crowdsourcing of reviews, then the editor to obtain *Q*_*r*_ as the average value of both the regular and the online reviewers comments. However, changes will not be made in the selected values of variables and parameters, only in the relations between them. Since the submodels can be parameterized independently, modifications into any of them do not affect the function of the other. The model will then be able to produce estimates for outputs of systems that have never been implemented in real life. One scenario is how many articles could be published and how fast by an alternative system under the same conditions as the conventional system.

A limitation of our simulations is the use of one-dimensional *Q* scores. A multidimensional version would treat separately factors such as importance, novelty and controversy arising from the manuscript. For this first exploration, a one-dimensional *Q* score variable was considered as a satisfactory proxy of all the quality dimensions that a manuscript incorporates. Another limitation is that the peer-review process did not capture the full complexity of interactions as occurs in real life. In next versions of the model, we could increase the complexity of the peer-review process and compare the impact that cooperation and competition between authors, reviewers and editors might have on the system. For example, we could examine in more detail scenarios of conflict of interest and competition for priority between authors and reviewers. We could also make authors spend more resources in the revisions of the paper if the evaluation from the reviewers is closer to the rejection than the acceptance threshold. Furthermore, since reviewers benefit in terms of knowledge from reviewing papers, their rewards could be connected to the *Q* score of the respective paper. An additional limitation is that our model represents a simplified abstraction of the reality. Arbitrary choices are at some point necessary in order to model real life systems, especially when empirical data are absent. However, to address this limitation, we performed extensive sensitivity analyses, whereby we explored the behavior of the model under several scenarios. A final limitation is that our calibration does not include open-access journals, which can have very different characteristics from traditional style journals. Adding more data, from open-access journals, will increase the accuracy of our calibration measures for scientific publication.

## Conclusion

We have developed an ABM that simulates the complexity of scientific publication and peer review and parameterized to fit to certain empirical data coming the biomedical literature. This model produced outputs for both published and unpublished articles. After structural changes to its submodels, we could simulate alternative peer-review systems. The alternative systems that will be produced, depending on the structural changes implemented, will not necessarily be calibrated to the data we used to calibrate the base model. This situation will produce deviations to the measured outputs that will allow us to compare the alternatives to the base system. These comparisons could help highlighting the most promising interventions that may to improve the system and place them under real-life examination.
